# Determinants of Health Care Needs in Relation to Vision Correction among Adolescents in the United Arab Emirates: A Cross-Sectional Study

**DOI:** 10.3390/ijerph20166547

**Published:** 2023-08-08

**Authors:** Faryal Maniyali, Otto Sanchez, Efrosini Papaconstantinou, Caroline Barakat

**Affiliations:** Faculty of Health Sciences, Ontario Tech University, Oshawa, ON L1G 0C5, Canada

**Keywords:** vision correction, adolescent, self-reported, UAE, prevalence

## Abstract

Background: Uncorrected refractive error has been suggested to affect children’s development, educational performance, and socialization. Sociodemographic and environmental differences among individuals may impact their accessibility to utilizing appropriate services, impacting their vision-dependent activities. Methods: Guided by the population health framework, this retrospective study assessed the prevalence of self-reported vision correction needs and its determinants for a sample of adolescents (n = 6363) from the United Arab Emirates (UAE) aged 13 to 20 years between 2007 to 2009. Results: The findings suggest a relatively high prevalence of self-reported vision correction needs (26.8%), with among 24.8% males and 31.7% among females. Factors that were significantly associated with vision correction needs included age, biological sex, location of residence (emirate), nationality, parental education and employment level, household financial status, screen time use, visiting an eye specialist in the past year, and daily functional capacity. Conclusion: Reporting the vision correction needs of the adolescent population and identifying its determinants may help identify and resolve modifiable barriers to accessing the appropriate vision care resources. Further research in assessing the type of refractive error, potential genetic and environmental factors, and vision care services in each emirate may help decision-makers set appropriate policies to improve the overall quality of eye health.

## 1. Introduction

Vision impairment due to uncorrected refractive error is the leading ocular problem affecting all age groups and the most easily preventable cause of disability [1,2,3]. Refractive error is known as the first cause of vision impairment and the second cause of visual loss worldwide [4,5]. Globally, 43% of vision impairment is ascribed to refractive errors, with an increase in prevalence from 84.8 million to 116.3 million between 1990 and 2015. There are three main forms of refractive error, which are astigmatism (non-spherical curvature of the cornea), myopia (near-sightedness), and hyperopia (far-sightedness) [1]. A global collection of population-based surveys and school-based surveys for the Refractive Error Study in Children (RESC) identified myopia as a major public health concern [6,7,8,9,10,11,12,13,14,15,16,17,18].

Impaired vision has been suggested to affect children’s cognitive and motor development, educational performance, and socialization, since they continue to receive increasing visual tasks as they progress in school [19,20]. Visual impairment due to refractive error negatively affects economic advantages, employability, physical safety in work and play, and general quality of life [6]. The global economy loses $269 billion annually due to lost productivity from URE (Smith et al., 2009) [21]. Among children aged 5–15 years worldwide, 12.8 million have been estimated to have vision impairment due to refractive error, accounting for 0.97% [22]. Correction of refractive errors among school students requires early detection, screening, and assistance, and is often treated with corrective lenses or refractive surgery [23]. Childhood and adolescence is a critical period of eye development and approximately 80% of student learning occurs through visual tasks (reading, writing, board work, using a computer) [24]. This demographic may have a higher sensitivity to environmental factors and a high risk of suffering from vision impairment from uncorrected refractive error, leading to reduced student academic and social participation [19]. However, socioeconomic factors such as poverty and limited ability to access treatment may influence the correction of refractive errors. Uncorrected refractive errors can also contribute to the individuals’ and their respective families’ socioeconomic status [23]. The WHO recommends the integration of vision screening and refractive services for school students and prioritizes the control of blindness in children in the “Vision 2020—the Right to Sight” program [25]. This is particularly important in areas where the integration of vision screening and refractive services is absent. Therefore, it is important to examine differences in the prevalence of uncorrected refractive error in relation to socioeconomic status, ethnic groups, and geographic regions, and to identify determinants of health care needs in relation to vision correction in the school-age population. Identifying determinants of vision care needs and who is at most risk can assist in implementing interventions with respect to visual aid services for students to help them academically, socially, and functionally improve in their developing years.

This study presents a scoping literature search that was conducted to review the prevalence of health care needs for vision correction in relation to socioeconomic and demographic factors, as well as lifestyle behaviors. An electronic search of PubMed, Ovid Cochrane, and Web of Science database sources were used. Keywords from the research topic were used to focus on articles pertaining to adolescents with VI-RE, including prevalence, determinants and/or associations, and vision impairment due to refractive error. Articles that were excluded included articles older than 10 years and not written in the English language, subject matter that focused on VI due to other reasons, a population other than adolescents, and studies focusing on a vulnerable or diseased population. Findings from the articles were summarized in a table with the characteristics of the study, population, prevalence of VI-RE, and associating factors along with their direction. Out of the ten included studies, there were three studies that were conducted in Latin America (Mexico, Paraguay, and Colombia), three studies were conducted in Africa (Nigeria, Somalia, and South Africa), one study was conducted in China, two studies were conducted in the Middle East (Saudi Arabia and the United Arab Emirates), and one study was a multicenter global systematic review. With respect to prevalence and determinants of vision correction needs among adolescents at the global level, the prevalence of myopia was reported in several national-based studies, with common determinants of increasing age [24,26,27,28], sex (with a higher prevalence among females) [24,27,28,29], higher socioeconomic status in terms of higher education and urban living [26,27,30,31], and lower outdoor activity [22]. One study assessed the self-reported health outcomes of vision correction needs and found a complaint of blur of 12.7%, where the prevalence of refractive error was 15.70% and vision impairment was 7.60% [32]. Limitations in previous research include the inclusion of participants in a wide range of age groups, research based on different scales and settings (e.g., school-based vs. national-based studies; urban vs. rural settings), varying methods of assessing vision impairment due to refractive error, and limited studies noting self-reported vision correction needs. Limited studies on self-reported health care needs in relation to vision correction are a barrier to identifying individuals that require visual aid in order to function in relation to their daily personal and academic activities. Adolescent self-reported health care needs can help examine environmental and lifestyle barriers linked to vision correction, and may help provide more grass-root-level solutions to optimally and efficiently treat those with vision impairment due to refractive error, thus enabling a better academic, social, and personal future for our youth.

The United Arab Emirates (UAE) is a country comprising seven emirates located in the southeast region of the Arabian Peninsula [33]. The population of the UAE has greatly increased over the past four decades [34]. This is mainly due to the high net inward migration of expatriate workers, creating a population structure of 11% Emiratis and the remaining expatriates of different nationalities [34,35]. Health care is provided for all nationals, but non-nationals are expected to secure mandatory health insurance often based on medical coverage from employment [36]. This greatly impacts the equal and affordable accessibility to vision care services to children under the age of 18 years based on the location of residence and household income. Moreover, further research is needed to explore school-based health services in both public and private schools within the UAE. School health services in Dubai are also categorized into public and private sectors, where the Ministry of Health (MoH) oversees public schools and the Dubai Health Authority (DHA) oversees the private schools [37]. In Dubai, there are 81 public schools and 186 private schools, where only private schools have a Schools and Educational Institutions Health Unit (SEIHU) from the Primary Health Care Services Sector (PHCSS) of the DHA that help conduct health assessments and support. The school health team helps develop guidelines, policies, and training to improve child health at private schools, where each school has a nurse and a doctor. There are six out of one hundred and eighty-six (3.2%) DHA-supervised special needs schools for children with special education needs (SEN), which include children dealing with eye disorders as a primary or secondary cause. Among public schools in Abu Dhabi, the Comprehensive School Screening Program, part of the Preventative Screening for Children launched by HAAD in 2010, includes vision screening among other components for early recognition of health problems in children [38]. It is important to note that the DHA and HAAD were not established from 2007 to 2009, which is the study timeline assessed in this study. However, there are no publications found to refer to the school-based vision screening process amongst the entire UAE in both public and private schools, and no self-reported concerns of students having difficulty seeing, pursuing vision tasks, and/or accessing resources for vision correction. The potential discrepancies in school-based health care services and assessments between public and private schools, with unknown vision screening services within emirates, may result in gaps in physician-diagnosed or self-reported vision impairment requiring vision correction.

The following study aims to fill in the knowledge gaps by answering the following questions:

(1) What is the prevalence of health care needs in relation to vision correction among adolescents from the UAE based on self-reported symptoms?
(2) What sociodemographic, behavioral, and physical factors are associated with health care needs in relation to vision correction among adolescents from the UAE?
(3) Are the health care needs in relation to vision correction affecting the functional capacity of high school students in the UAE?
(4) How do the prevalence and determinants of health care needs in relation to vision correction among high school students in the UAE compare to the existing evidence?

Thus, the research objectives of this study were: (1) Assess the prevalence of self-reported health care needs in relation to vision correction among adolescents from the UAE. (2) Assess if there are significant associations between vision correction needs and each of the following factors: socioeconomic status, physical and lifestyle behaviors, and affecting functional capacity for the UAE adolescent population.

## 2. Materials and Methods

### 2.1. Study Design and Ethical Approval

Guided by the population health framework, a retrospective quantitative cross-sectional design was conducted to examine the prevalence of self-reported health care needs, and to identify significant associations between sociodemographic and behavioral factors and self-reported health care needs in relation to vision correction, for the UAE [39]. This was carried out using data from the National Study of Population Health in the UAE (NSPHUAE) (2007–2009) research program developed and conducted by researchers at Zayed University in collaboration with the UAE Ministry of Education. The program involved administering a comprehensive cross-sectional health survey to adolescents across public and private schools in the seven emirates of the UAE. This research study was approved by the Ontario Tech University Ethics Committee (REB file # 16553).

### 2.2. Population and Sampling

The NSPUAE program presents a cross-sectional survey administered to 6363 adolescents from 147 schools aged 13 to 20 years attending public and private schools in seven emirates of the federation including nine educational zones [40]. This was a randomly selected stratified sample using school enrolment from the UAE Ministry of Education (2005–2006 for private schools and 2006–2007 for public schools). Social workers, employed by the UAE Ministry of Education and trained by researchers through workshops, conducted the developed survey on students from three randomly selected classes (grades 10, 11, and 12) from each participating school through a random stratified sampling strategy with parental consent. The completed two-component self-reporting questionnaires were then collected by the social workers.

### 2.3. Measurement Tools

The first component was completed by adolescent participants, which comprised data on sociodemographic information, recreational behaviors such as smoking and physical activity, medical conditions, and symptoms with respect to respiratory health [41]. The second component was completed by adolescents at home with parental assistance, which comprised data on residential and neighborhood characteristics, and previous residence information.

### 2.4. Study Variables

The dependent variable is a two-level nominal (categorical) variable of yes or no to seeing well enough to read without the use of glasses. The independent variables include 19 potential determinants of health in relation to vision correction (Table 1).

### 2.5. Data Analysis

The dataset provided was inspected and analyzed using IBM SPSS version 26. Univariate analyses were conducted to describe the patterns for continuous data, including mean, mode, median, standard deviation, and the overall summary of the data [42]. Categorical and nominal variables were placed in a frequency table to showcase the prevalence of each value within the variable. The continuous variables of age and the number of individuals in a residence were analyzed with the descriptive outputs of the mean and standard deviation of the variable.

Bivariate analyses were conducted to assess associations between independent variables and the outcome of the self-reported health care need in relation to vision correction. The nominal/categorical variables were analyzed using Pearson chi-square tests. Continuous variables including age and the number of individuals in a residence underwent independent *t*-tests. The results were expressed using chi-square (X^2^) and *p*-values for categorical variables, and t-values and *p*-values for the *t*-test for continuous variables (where a *p*-value of less than 0.05 was considered a significant association). *p*-values of 0.1 and less were also included to be added in the multivariate analysis.

Statistically significant variables with a *p*-value of less than 0.1 in the chi-square test for categorical variables and a *p*-value of less than 0.1 in the *t*-test for continuous variables were entered into the multivariate analysis. As the dependent variable is a binary variable of yes or no for “seeing well enough to read without the use of corrective lenses”, a binary logistic regression analysis was conducted to identify determinants of vision correction needs. In addition to the model test using a significant *p*-value of less than 0.05 to potentially represent a significant improvement in fit relative to the null (only intercept) model, a Hosmer and Lemeshow test was also included to use non-significance as an indicator of goodness of fit, to check how well the data fit the models. The binomial logistic regression estimates the probability of the student reporting not seeing well enough to read without the use of corrective lenses. Therefore, it is important to use this method to predict if the cases are correctly classified from the independent variables by assessing the effectiveness of the predicted grouping/classification against the observed classification. This was carried out using the classification table. The indication of fit of the model through specificity and sensitivity was observed using the percentage of correct classification of the predicted and observed values of the dependent variable. For the statistically significant variables, the odds ratio, confidence interval, and *p*-value (including *p*-values of 0.05 or below) were presented. These values indicated the likelihood of a response value being associated with the self-reported vision correction needs compared to the reference response value.

To assess the daily functional capacity of students needing vision correction based on their use of vision care services, a flow chart of students reporting to need vision correction was formed within each emirate. Among these students, the percentage of students reporting to visit an eye specialist in the past 12 months was noted, followed by the percentage of students from each category reporting their ability to handle daily work/school responsibilities.

## 3. Results

### 3.1. Findings from the Descriptive Analysis

Table 2 includes a descriptive analysis for each categorical variable with the frequency of each value and its proportion. It also includes the mean and standard deviation for continuous variables. The findings are further categorized into biological sex-based descriptive analysis. From the questionnaire, 28.6% of the students responded that they could not “see well enough to read without the use of corrective lenses”, indicating needing vision correction, with 24.8% among males and 31.7% among females.

### 3.2. Findings from the Bivariate Analysis

Table 3 describes the variables found to be associated with vision correction needs. Table 4 describes the associations in the biological sex-based analysis.

#### 3.2.1. Demographic Factors

- Whole sample

Among the demographic factors, significant associations were found between age (t = −1.72, *p* = 0.008), biological sex (X^2^ = 35.4, *p* < 0.001), the location of residence (emirate) (X^2^ = 916.7, *p* < 0.001), and nationality (X^2^ = 58.6, *p* < 0.001) with needing vision correction. More females were found needing vision correction than males. Students from Fujairah reported to have the highest need for vision correction. Students with a nationality from South Asia were found to have the highest percentage of needing vision correction.

- Biological sex-based analysis

In the biological sex-based analysis, significant associations were found between age (female: t = −0.59, *p* = 0.071), the location of residence (emirate) (male: X^2^ = 326.5, *p* < 0.001; female: X^2^ = 914.4, *p* < 0.001), and nationality (male: X^2^ = 11.37, *p* = 0.045; female: X^2^ = 49.75, *p* < 0.001) and needing vision correction. More females from Abu Dhabi and Dubai reported needing vision correction than males, yet for the other emirates, more males reported needing vision correction than females. More females reported needing vision correction than males from all categories of nationalities.

#### 3.2.2. Socioeconomic Factors

- Whole sample

Regarding socioeconomic factors, associations were found between needing vision correction and paternal educational level (X^2^ = 24.9, *p* < 0.001), maternal educational level (X^2^ = 24.5, *p* < 0.001), paternal employment status (X^2^ = 21.0, *p* < 0.001), maternal employment status (X^2^ = 9.7, *p* = 0.024), residential ownership (X^2^ = 19.4, *p* < 0.001), and household monthly income (X^2^ = 9.1, *p* = 0.003). More students reported needing vision correction whose parents had completed high school, were private employees, rented their home, and had a household monthly income of 15,000 AED and above compared to those whose parents had not completed high school, were employed in other categories or were not employed, owned their home, and had a household monthly income of less than 15,000 AED.

- Biological sex-based analysis

In the biological sex-based analysis, associations were found between needing vision correction and paternal educational level (male: X^2^ = 7.10, *p* = 0.008; female: X^2^ = 16.89, *p* < 0.001), maternal educational level (male: X^2^ = 8.56, *p* = 0.003; female: X^2^ = 15.72, *p* < 0.001), paternal employment status (male: X^2^ = 6.05, *p* = 0.109; female: X^2^ = 15.98, *p* = 0.001), maternal employment status (female: X^2^ = 9.29, *p* = 0.026), residential ownership (male: X^2^ = 6.89, *p* = 0.009; female: X^2^ = 13.78, *p* < 0.001), and household monthly income (female: X^2^ = 5.72, *p* = 0.017). There were more females than males reporting needing vision correction among both levels of parental education, all levels of parental employment status, with the highest prevalence among parents who were private employees, and had a household monthly income of 15,000 AED and above.

#### 3.2.3. Behavior and Lifestyle Factors

- Whole sample

While the use of near-work devices and outdoor activity were not significantly associated with vision correction needs, screen time was associated with vision correction needs (*p* = 0.116), with the highest prevalence of needing vision correction among those using screen time for a moderate number of hours.

- Biological sex-based analysis

In the biological sex-based analysis, significant associations were found between needing vision correction and mobile phone use per day (female: X^2^ = 9.81, *p* = 0.007) and outdoor physical activity (female: X^2^ = 2.80, *p* = 0.094). For males, as the number of hours of mobile phone use per day increased, the prevalence of vision correction needs decreased, with the opposite trend for females. With respect to outdoor activity, there was a larger and opposite directional difference between males and females. Among males, a higher prevalence of vision correction needs was found among those pursuing no outdoor activity, and among females, a higher prevalence of vision correction needs was found among those pursuing at least one outdoor activity.

#### 3.2.4. Health Care and Functional Capacity

Vision correction needs were significantly associated with visiting an eye specialist in the past year (X^2^ = 394, *p* < 0.001), and having a ‘poor/fair’ handle on school/work responsibilities (X^2^ = 17.5, *p* < 0.001), which was consistent among males and females, with a higher prevalence among females.

### 3.3. Findings from the Multivariate Analysis

Binary logistic regression analysis suggested that significant determinants of vision correction needs include nationality, the location of residence (emirate), screen time use, and visiting an eye specialist in the past 12 months (Table 5). For males, age, the location of residence (emirate), nationality, visiting an eye specialist in the past 12 months, and handling daily school/work responsibilities were significant determinants of vision correction needs. For females, the location of residence (emirate), nationality, maternal education, and visiting an eye specialist in the past 12 months were significant determinants of vision correction needs (Table 6).

- Whole sample

Overall, students living in Dubai (OR: 2.746, 95% CI: 2.045, 3.689, *p* < 0.001), Fujairah (OR: 9.533, 95% CI: 6.307, 14.410, *p* < 0.001), and UAQ (OR: 7.570, 95% CI: 3.779, 15.163, *p* < 0.001) were more likely to report needing vision correction than those living in Abu Dhabi. Students noting their nationality as being from South East Asia were more likely to report needing vision correction than those of UAE nationality (OR: 1.996, 95% CI: 1.303, 3.058, *p* = 0.002). Students who reported a moderate number of hours of screen time in a day were more likely to report needing vision correction than those who reported minimal hours of screen time in a day (OR: 1.401, 95% CI: 1.068, 1.838, *p* = 0.015). Students who had visited an eye specialist in the past 12 months were more likely to report needing vision correction (OR: 4.038, 95% CI: 3.051, 5.346, *p* < 0.001).

- Male analysis

For males, students of increasing age were less likely to report needing vision correction. Students residing in Fujairah (95% CI: 18.849, 364.023, *p* < 0.001), Sharjah (95% CI: 1.013, 2.138, *p* = 0.043), and UAQ (95% CI: 3.348, 88.600, *p* < 0.001) were more likely to report needing vision correction than those from Abu Dhabi. Students from other GCC countries were more likely to report needing vision correction than those from the UAE (95% CI: 1.111, 4.175, *p* = 0.023). Students who had visited an eye specialist in the past 12 months were more likely to report needing vision correction than those who did not visit an eye specialist in the past 12 months (95% CI: 2.529, 6.620, *p* < 0.001). Students noting poor to fair ability to handle daily school/work responsibilities were less likely to report needing vision correction than those noting good to excellent in handling daily responsibilities (95% CI: 0.264, 0.737, *p* = 0.002).

- Female analysis

For females, students residing in Dubai (95% CI: 4.305, 9.356, *p* < 0.001), Fujairah (95% CI: 4.161, 10.734, *p* < 0.001), and UAQ (95% CI: 2.817, 13.493, *p* < 0.001) were more likely to report needing vision correction than those residing in Abu Dhabi. Students from South East Asia were more likely to report needing vision correction than students from the UAE (95% CI: 1.027, 3.368, *p* = 0.041). Students whose mothers completed high school were more likely to report needing vision correction than those whose mothers did not complete high school (95% CI: 1.015, 2.224, *p* = 0.042). Students who had visited an eye specialist in the past 12 months were more likely to report needing vision correction than those who did not visit an eye specialist in the past 12 months (95% CI: 2.577, 5.434, *p* < 0.001).

### 3.4. Assessing the Daily Functional Capacity—Flow Chart

Among the whole sample, a flow chart was made to indicate the following:

(1) Number of adolescents reporting needing vision correction in each emirate;
(2) Within each emirate, how many adolescents needing vision correction visited (yes)/did not visit (no) an eye specialist in the past year;
(3) Among adolescents visiting (yes)/not visiting (no) an eye specialist in the past year, how many reported good to excellent or poor to fair academic functional capacity.

**Figure 1:**
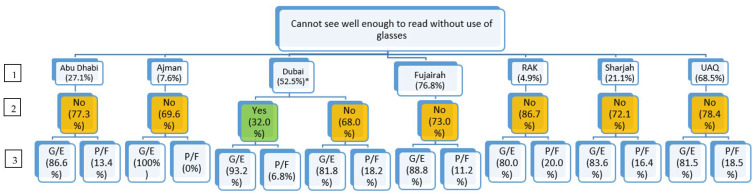
Emirate-based analysis of vision correction needs with health care access and functional capacity. Legend: 1. Emirate-based prevalence of adolescents reporting needing vision correction. 2. Visiting an eye specialist in the past 12 months. 3. Handling daily work. G/E = good to excellent. P/F = poor to fair. * *p* < 0.05.

Among students reporting needing vision correction, the majority did not report visiting an eye specialist in the past 12 months (Figure 1). This was found to be the highest among students from RAK (86.7%). There were five emirates where the percentage of participants who reported poor to fair daily functional capacity was greater among those who did not visit an eye specialist in the past year compared to those who visited an eye specialist in the past year, with a significant association within the emirate of Dubai (18.2% vs. 6.8%) (X^2^ = 6.22, *p* = 0.013).

## 4. Discussion

### 4.1. Prevalence and Determinants of Self-Reported Vision Correction Needs

The results indicate that 28.6% of the adolescents responded needing vision correction, which is lower than the global prevalence of vision impairment ascribed to refractive errors as 43% [4]. The relatively higher percentage of students with the self-reported health care need for vision correction among adolescents in this study may be due to the lack of prior academic health screening, low socioeconomic status in the form of household monthly income, and reduced outdoor activity due to weather conditions. The lack of timely screening of vision impairment due to refractive error among adolescents in the UAE may have resulted in a significant percentage of students reporting needing vision correction in their adolescent years. While the Ministry of Health (MoH) oversees public-school health services and the Dubai Health Authority (DHA) oversees private schools, the DHA was only launched in March 2008, including the Schools and Educational Institutions Health Unit (SEIHU) from the Primary Health Care Services Sector (PHCSS) of the DHA that help conduct health assessments and support [37]. In Abu Dhabi, the Comprehensive School Screening Program, part of Preventative Screening for Children, was launched by the Health Authority of Abu Dhabi (HAAD) in 2010 [38]. Therefore, a comparison cross-sectional survey among adolescents in the UAE in the current timeframe may help identify changes in health outcomes and potential remaining barriers. The majority of the adolescents were found to have a household monthly income of less than 15,000 AED. Since vision care requires an employment insurance plan dependent on an employee’s salary and designation, families falling under the low-income category may not be able to provide access or continued access to vision care for their children in their childhood and/or adolescent years [43]. This finding is consistent with 91.9% of adolescents that had not visited an eye specialist in the past year. Therefore, it is suggested to provide vision care services to school-based children and adolescents as part of standard health care coverage from the Ministry of Education and Ministry of Health across all emirates, irrespective of household monthly income. While pursuing at least one outdoor activity was not shown to be significantly associated with self-reported needs for vision correction in this study, the warmer climate in the UAE may pose contextual barriers to pursuing outdoor activities, as suggested among women in a Qatari study [44,45]. Further research assessing the vision correction needs among adolescents in different climates in the UAE based on outdoor temperatures may help identify any associations between weather, pursuing outdoor activity, and vision correction needs.

Both bivariate and multivariate male analyses suggested that age is an associating factor of vision correction needs, consistent with existing evidence. Previous studies in the literature found older participants to present with hyperopic astigmatism and a gradual increase in myopia, and an increased burden was found with increasing age among both biological sexes [24,27,28,29]. Adolescents of all nationalities may be required to learn English and be placed in pressurizing academic near-work settings, especially in their adolescent years to achieve high grades to enter one of UAE’s prominent universities known to guarantee lucrative employment opportunities. Adolescents have shown a positive association between near work and myopia and astigmatism, predominantly seen to be increasing among adolescents from Asian countries in the past three decades [1,46,47,48,49]. However, for the male analysis, the inverse association requires further research in incorporating the type of vision impairment due to refractive error using an ophthalmic examination along with self-reported vision correction needs. This additional assessment may help better understand if there are differences in the prevalence of myopia, hyperopia, and astigmatism between males and females between younger and older adolescents.

The higher percentage of females reporting needing vision correction is consistent, with five studies included in the literature review noting significant differences between biological sex [24,27,28,29]. In these five studies, female participants appeared to have a higher association with refractive error with respect to myopia and higher disease burden due to uncorrected refractive error [24,27,28,29,50]. Females were found to have a lower distribution of paternal employment, lower distribution of low household monthly income, and higher distribution of owning their residence. More females were found to spend 5 or more hours a day watching television, and significantly more females were found to visit an eye specialist than males, which suggests further research in identifying genetic predispositions and socio-environmental factors.

Both bivariate and multivariate analyses found the location of residence (emirate) to be associated with vision correction needs. Adolescents residing in Fujairah had the highest percentage of participants reporting needing vision correction, followed by UAQ, Dubai, Abu Dhabi, and Sharjah, and the lowest percentage was in Ajman and RAK. Fujairah and UAQ had the lowest percentages of population distribution of residence (7.0% and 2.2%, respectively) and the lowest distribution of government and private hospitals, with Fujairah having two government hospitals and one private hospital, and UAQ having one government hospital and no private hospitals [36,51,52]. With limited access to a hospital and/or government-funded primary health care clinics (PHC) that provide eye examination services due to a relatively lower population size, adolescents living in Fujairah and UAQ may be at a disadvantage in terms of timely vision screenings than those living in other emirates.

Findings from both bivariate and multivariate analyses suggested that adolescents from South East Asia had the highest number of students needing vision correction, followed by adolescents from Western countries, Middle East countries, other GCC countries, and, lastly, those noting no nationality or “other”. There were more females who reported needing vision correction than males from all categories of nationalities with the greatest difference among females from South East Asian countries. The findings are consistent with studies conducted in South East Asian countries noting a high prevalence of myopia among children and adolescents in the literature [13,24,53,54]. However, these findings are in contrast to the low prevalence of hyperopia and astigmatism found in studies comparing different nationalities/ethnicities, where the lowest prevalence was found among students from South East Asian nationalities [4]. This may suggest a higher prevalence of myopic self-reported vision correction needs among adolescents in the UAE. Adolescents of South East Asian nationalities having parents with higher educational levels may result in parental influence on increased academic near work and decreased outdoor activity, which may be positively associated with vision impairment due to refractive error, especially in terms of myopia [55].

Higher paternal and maternal educational levels in terms of completing high school were found to be significantly associated with higher self-reported vision correction needs, with a higher distribution among females. This is consistent with existing evidence where higher levels of parental education have been reported to have a positive correlation with the prevalence of vision impairment due to refractive error among children and adolescents [17,19,56,57]. Increased parental pressure of attaining similar or higher educational levels, especially in urban environments where employment levels may be majorly dependent on one’s educational status, may be a potential factor in the association between parental education and vision correction needs. Moreover, potential cultural differences of influencing females to engage in fewer outdoor activities and increased near work, may be potential factors in the association between parental education and vision correction needs.

Paternal and maternal employment statuses were found to be significantly associated with self-reported vision correction needs, with a mildly increased percentage for adolescents whose parents were private employees and a higher distribution among females. The increased percentage among those whose parents were private employees appears consistent with the previous literature, showcasing a higher prevalence of myopia among adolescents of parents with professional occupations [58,59]. Therefore, the findings suggest employment status is positively associated with the prevalence of visual impairment. However, the overall similar distribution of self-reported vision correction needs between different types of employment categories potentially demonstrates a fairly uniform educational system and curriculum among public and private schools in the UAE.

Household financial status in terms of lack of residential ownership and a higher household monthly income (15,000 AED or above) was found to be significantly associated with self-reported vision correction needs, with a higher distribution among females. These findings are consistent with those from the literature review, including the study based in Australia where children with parental home ownership were less likely to have visual impairment than those without parental home ownership [59]. The findings are also consistent with the studies based in Korea and Guangzhou, China, with an increased prevalence of myopia among children from higher family incomes [58,60]. Moreover, the current findings align with the review by Yang et al. [24], who found the DALY rates, or global disease burden of uncorrected refractive error (URE), to be highest in high-income regions [24].

The results from both the bivariate and multivariate analyses found screen time use to be significantly associated with self-reported needs for vision correction. There was an overall similar distribution of adolescents reporting vision correction needs, with the highest among those with a moderate number of screen time hours (5–9 h). While the use of mobile phones was found to be significant among females, the type of mobile phones around 2007–2009 commonly used among the adolescent population included basic functions, and thus may not be solely associated with visual use of the phone as a near task. While these results are in contrast to the study based in Riyadh, Saudi Arabia, which did not find a significant association between frequency and time spent on homework or electronic devices and having an RE [22], they coincide with studies that suggested a positive association between near work and myopia, astigmatism, and a higher prevalence of near work and VI-RE among children and adolescents from South Asian countries than those from the UK and the USA [1,46,47,48].

### 4.2. Visiting an Eye Specialist in the Past Year

Visiting an eye specialist in the past 12 months was found to be significantly associated with self-reported vision correction needs, with a higher distribution among females. This finding may be indicative that adolescents visiting an eye specialist may be addressing their self-reported vision correction needs and potentially be finding solutions through spectacles, contact lenses, or other means of treating their visual impairment. The increased distribution of females is in contrast with the study based in the United States, which found that among those with similar health care needs, women were found to make fewer physician visits than males in the older population [61]. However, the results are consistent with the findings in the Portugal-based study, where women above 18 years of age had higher health care use than men, including specialist visits even when aligning for similar morbidity and socioeconomic differences [62].

### 4.3. Daily Functional Capacity

The bivariate analysis found more adolescents needing vision correction to report poor to fair academic performance than those reporting good to excellent academic performance. However, in the multivariate analysis, male adolescents that responded to having poor to fair ability to handle daily school/work responsibilities were less likely to report needing vision correction. There was a higher percentage of adolescents who identified needing vision correction and not visiting an eye specialist in the past year to report having poor to fair daily functional capacity in handling daily academic tasks than those visiting an eye specialist in UAQ followed by Dubai, Sharjah, Abu Dhabi, and Fujairah. The results of this study coincide with the suggestions by the WHO and the study by Rudnicka et al. [19], suggesting impaired vision affects children’s educational performance due to increased visual tasks with academic progression as well as sociodemographic differences, potentially impacting their daily vision-dependent activities [19,20]. Further research on exploring the biological sex-based differences in vision correction needs and daily functional capacity, affordability and accessibility to access vision care services within each emirate, and other potential barriers for these adolescents to not visit an eye specialist need to be explored to further understand the reasoning behind their relatively lower daily functional capacity.

### 4.4. Limitations

This study conducted a retrospective analysis of a dataset based on a cross-sectional survey conducted between 2007 to 2009. The findings from this study reflect the prevalence and determinants of vision correction needs during those years, which can assist in comparing further research on the current situation among adolescents in the UAE with the addition of school-based vision screening services.

The measurement instruments regarding screen time inquire about the number of hours spent per day on television, computer/video games, and mobile phones. However, the location of use (school vs. home) and learning mechanism of the school (more book vs. screen oriented) were not indicated in the dataset. Further research on the learning environment and the location of screen time use may help better determine the reasoning to use various screen devices.

As the questionnaire conducted was using self-reported responses, there is a risk of low response, subjective recall bias, and an inability to verify their responses.

The relatively higher levels of missing values for certain variables, including parental education levels, parental employment status, household monthly income, location of residence (emirate), and residential ownership, indicate gaps in the completion of various socioeconomic household-based sections of the questionnaire by the participants that may be due to an inability to recall the appropriate response or unwillingness to share the information.

This study is based on a cross-sectional survey; therefore, the exposure and outcome are analyzed at the same time and the temporal association of determinants and vision correction needs is not measurable.

The dependent variable in this study is asking the participants to self-report their ability to see well enough to read without the use of glasses. While this provides a direct indication of the participants’ need for vision correction, it does not indicate if they have glasses or other forms of vision correction, and does not refer to an objective ophthalmic examination to identify the type of vision impairment due to refractive error.

The location of residence is based on the emirate, which does not identify the number of vision care services near the participant’s residence and their accessibility to accessing those services. As there were potential confounding variables that may involve interference between independent variables, a binary logistic regression was used to control confounding variables.

## 5. Conclusions

VI-RE has been suggested to negatively affect academic performance and overall quality of life, in particular among the adolescent age group where academic visual tasks increase with age, requiring early screening and assistance in accessing vision correction resources, and where detection and access may be influenced by sociodemographic and environmental factors. Reporting the symptomatic complaints of the adolescent population regarding vision correction needs and identifying determinants may help determine which participants require vision correction and help the population in resolving modifiable barriers to accessing the appropriate vision care resources. This study assessed the prevalence and determinants of self-reported vision correction needs among the adolescent population aged 13 to 20 years in the UAE using a retrospective quantitative data analysis between 2007 to 2009. The results confirmed that age, biological sex, the location of residence (emirate), nationality, parental education and employment level, household financial status, screen time use, visiting an eye specialist in the past year, and daily functional capacity were associated with self-reported vision correction needs. However, further research is suggested to compare these findings with a recent survey among the same-aged demographic in the UAE, having ophthalmic examinations alongside self-reported outcomes to identify the type of VI-RE, biological sex-based and ethnic genetic predispositions, and sociocultural differences related to parental socioeconomic status. Further research on exploring the number of vision care services in these emirates, affordability and accessibility to access these services, and other potential barriers for these adolescents to visit an eye specialist need to be explored to further understand the reasoning behind their relatively lower daily functional capacity. It may help future researchers determine patterns of eye care utilization by this population, vision screening procedures and policies in academic settings, and academic and social performance of this population, thus helping decision-makers set appropriate plans, policies, and strategies to prevent visual impairment and improve overall eye health.

## Figures and Tables

**Table 1:** List of 19 potential health determinants assessed in the survey.

Type of Health Determinant	Variables
Demographic	1. Sex
	2. Age
	3. Ethnicity (Nationality): i. The UAE ii. Other Gulf Cooperation Council (GCC) countries (Kuwait, Kingdom of Saudi Arabia, Oman, Qatar, Bahrain, and Yemen) iii. Other Middle East Arab countries (Lebanon, Syria, Jordan, Palestine, Iraq, North Africa, Yemen, and Somalia) iv. South East Asia (India, Pakistan, Bangladesh, Sri Lanka, Philippines, and Indonesia) v. Western countries (Europe, the USA, Canada, and Australia) vi. Others and no nationality
	4. Location of Residence (Emirate): i. Abu Dhabi ii. Ajman iii. Dubai iv. Fujairah v. Ras Al Khaimah (RAK) vi. Sharjah vii. Umm Al Quwain (UAQ)
Socioeconomic	5. Parental marital status. This has two categories: married and separated/divorced/widowed/others
	6. Paternal education
	7. Maternal education
	Both variables 6 and 7 have two categories: did not complete high school and completed high school
	8. Paternal employment status
	9. Maternal employment status
	Both variables 8 and 9 have four categories: government employee, private employee, self-employed, and not employed/retired
	10. Residential ownership (own, rent)
	11. Household income. This is a two-level categorical variable of those reporting a monthly household income of less than 15,000 AED (United Arab Emirates Dirham) and those reporting a household monthly income of 15,000 AED and above
	12. Number of individuals in residence
Behaviour and Lifestyle	13. Duration of use of television (hours/day)
	14. Duration of use of computer/video display games (hours/day)
	15. Duration of use of mobile phones (hours/day)
	For variables 13, 14, and 15, there are three categories of less than 1/NA hours, 1 to 4, and 5 or more
	16. Screen time use (combined use of television and computer/video games). There are four categories of minimal (0 to 5), mild (2 to 8), moderate (5 to 9), and high (10 or more)
	17. Pursuing at least one outdoor activity
Functional capacity	18. Ability to handle daily work or school responsibilities
Health care	19. Visiting an eye specialist in the past 12 months

**Table 2:** Descriptive sociodemographic, lifestyle behaviors, and functional capacity of a sample of adolescents living in the UAE (n = 6363).

Variable	n	Values	Male n (%)	Female n (%)	n Total (%)
Biological sex	6359	Male			2826 (44.4)
		Female			3533 (55.6)
Age	6144	(mean/SD)	17 years mean; SD: 1.228	16 years mean; 1.157	17 years mean; SD: 1.195
Nationality	6329	UAE	1233 (44.4)	1855 (53.0)	3127 (49.4)
		Other GCC countries	228 (8.2)	127 (3.6)	360 (5.7)
		Lebanon, Syria, Jordan, Palestine, Iraq, North Africa, Yemen, and Somalia	864 (31.1)	764 (21.8)	1632 (25.8)
		South East Asia (India, Pakistan, Bangladesh, Sri Lanka, Philippines, and Indonesia)	366 (13.2)	634 (18.1)	1006 (15.9)
		Europe, Canada, USA, and Australia	30 (1.2)	51 (1.6	82 (1.3)
		Others and no nationality	54 (1.9)	68 (1.9)	122 (1.9)
Location of Residence (Emirate)	5392	Abu Dhabi	1190 (52.4)	1244 (41.2)	2532 (47.0)
		Ajman	162 (7.0)	153 (5.0)	315 (5.8)
		Dubai	159 (7.0)	378 (12.5)	537 (10.0)
		Fujairah	79 (3.5)	301 (10.0)	380 (7.0)
		Ras Al Khaimah (RAK)	158 (7.0)	452 (15.0)	610 (11.3)
		Sharjah	495 (21.8)	404 (13.3)	899 (16.7)
		Umm Al Quwain (UAQ)	29 (1.3)	90 (3.0)	119 (2.2)
Parental marital status	6277	Married	2443 (88.6)	3073 (87.8)	5535 (88.2)
		Separate/Divorced, Widowed, Others	313 (11.4)	426 (12.2)	742 (11.8)
Paternal education level	5178	Did Not Complete High School	957 (42.5)	1192 (41.7)	2185 (42.2)
		Completed High School	1293 (57.5)	1668 (58.3)	2993 (57.8)
Maternal education level	5210	Did Not Complete High School	1144 (51.0)	1481 (51.1)	2664 (51.1)
		Completed High School	1097 (49.0)	1420 (48.9)	2546 (48.9)
Paternal employment status	5324	Government employee	1227 (52.1)	1236 (42.7)	2496 (46.9)
		Private employee	510 (21.6)	698 (24.1)	1222 (23.0)
		Self-employed	286 (12.1)	444 (15.3)	739 (13.9)
		Not employed/Retired	334 (14.2)	519 (17.9)	867 (16.2)
Maternal employment status	5453	Government employee	224 (9.4)	292 (9.8)	529 (9.7)
		Private employee	163 (6.8)	230 (7.7)	397 (7.3)
		Self-employed	67 (2.8)	77 (2.6)	147 (2.7)
		Not employed	1940 (81.0)	2388 (79.9)	4380 (80.3)
Residential ownership	5348	Own	1145 (48.4)	1544 (53.0)	2733 (51.1)
		Rent	1223 (51.6)	1371 (47.0)	2615 (48.9)
Number of individuals in residence	5068	(mean/SD)	8.60 (4.54)	8.88 (4.63)	8.74/4.59
Household monthly income	3940	Less than 15,000	1355 (76.2)	1545 (73.0)	2936 (74.5)
		15,000 and Above	423 (23.8)	571 (27.0)	1004 (25.5)
Television—h/day	6145	Less than 1/NA	595 (22.3)	747 (22.0)	1364 (22.2)
		1 to 4	1440 (54.0)	1756 (51.7)	3238 (52.7)
		5 or more	632 (23.7)	891 (26.3)	1543 (25.1)
Computer/Video-display games—h/day	6176	Less than 1/NA	774 (28.9)	1108 (32.4)	1908 (30.9)
		1 to 4	1191 (44.5)	1147 (43.3)	2703 (43.8)
		5 or more	713 (26.6)	831 (24.3)	1565 (25.3)
Mobile phone—h/day	6252	Less than 1/NA	1202 (44.2)	2356 (68.4)	3956 (57.6)
		1 to 4	827 (30.4)	697 (20.2)	1553 (24.8)
		5 or more	693 (25.4)	392 (11.4)	1103 (17.6)
Screen time—h/day	6061	Minimal	877 (33.4)	1163 (34.6)	2071 (34.2)
		Mild	758 (28.9)	898 (26.7)	1680 (27.7)
		Moderate	650 (24.8)	890 (26.5)	1552 (25.6)
		High	337 (12.9)	407 (12.2)	758 (12.5)
Outdoor physical activity	6359	No outdoor physical activity	536 (19.0)	1173 (33.2)	1709 (26.9)
		At least 1 outdoor physical activity	2290 (81.0)	2360 (66.8)	4650 (73.1)
Visiting an eye specialist in past year	6309	No	2496 (91.9)	2983 (84.9)	5555 (88.0)
		Yes	219 (8.1)	529 (15.1)	754 (12.0)
Handling daily work/school responsibilities	6272	Good to Excellent	2463 (89.4)	3033 (88.4)	5567 (88.8)
		Poor to Fair	292 (10.6)	399 (11.6)	705 (11.2)
See well enough to read without the use of corrective lenses	6392	Yes	2099 (75.2)	2399 (68.3)	4561 (71.4)
		No	694 (24.8)	1112 (31.7)	1831 (28.6)

**Table 3:** Bivariate analyses to assess associations between independent variables and self-reported health care needs in relation to vision correction.

Variable	Reference	Sees Well Enough to Read without Use of Corrective Lenses	Does Not See Well Enough to Read without Use of Corrective Lenses/Needed Vision Correction	*p*-Value
Biological sex (%)	Male	75.20	24.80	<0.001
	Female	61.30	31.70	<0.001
Age (years)		Mean: 17	Mean: 17	<0.05
Nationality (%)	UAE	73.20	26.70	<0.001
	Other GCC countries	76.30	23.70	<0.001
	Lebanon, Syria, Jordan, Palestine, Iraq, North Africa, Yemen, and Somalia	71.90	28.10	<0.001
	South East Asia (India, Pakistan, Bangladesh, Sri Lanka, Philippines, and Indonesia)	62.00	38.00	<0.001
	Europe, Canada, USA, and Australia	67.90	32.10	<0.001
	Others and No Nationality	81.00	19.00	<0.001
Location of Residence (Emirate) (%)	Abu Dhabi	72.90	27.10	<0.001
	Ajman	92.40	7.60	<0.001
	Dubai	47.50	52.50	<0.001
	Fujairah	23.2	76.80	<0.001
	Ras Al Khaimah (RAK)	95.10	4.90	<0.001
	Sharjah	78.90	21.10	<0.001
	Umm Al Quwain (UAQ)	31.50	68.5	<0.001
Parental marital status (%)	Married	71.60	28.40	
	Separated/Divorced/Widowed, Other	69.70	30.30	
Paternal education level (%)	Did not complete high school	75.80	24.20	<0.001
	Completed high school	69.50	30.50	<0.001
Maternal education level (%)	Did not complete high school	75.00	25.00	<0.001
	Completed high school	68.80	31.20	<0.001
Paternal employment status (%)	Government employee	73.80	26.20	<0.001
	Private employee	67.10	32.90	<0.001
	Self employed	70.20	29.80	<0.001
	Not employed/retired	74.00	26.00	<0.001
Maternal employment status (%)	Government employee	70.00	30.00	<0.05
	Private employee	65.80	34.20	<0.05
	Self employed	73.10	26.90	<0.05
	Not employed	72.70	27.30	<0.05
Residential ownership (%)	Own	74.70	25.30	<0.001
	Rent	69.20	30.80	<0.001
# of people in residence		Mean: 8.94	Mean: 8.24	
Household monthly income (%)	Less than 15,000 AED	73.20	26.80	
	15,000 AED and above	68.20	31.80	<0.05
Television—hrs/day (%)	Less than 1/NA	71.70	28.30	
	1 to 4	72.00	28.00	
	5 or more	70.00	30.00	
Computer/Video-display games—hrs/day (%)	Less than 1/NA	72.10	27.90	
	1 to 4	71.90	28.10	
	5 or more	69.90	30.10	
Mobile phone—hrs/day (%)	Less than 1/NA	71.10	28.90	
	1 to 4	71.30	28.70	
	5 or more	72.10	27.90	
Screen time—hrs/day (%)	Minimal	72.30	27.70	0.10
	Mild	72.80	27.20	0.10
	Moderate	69.30	30.70	0.10
	High	70.50	29.50	0.10
Outdoor physical activity (%)	No outdoor physical activity	71.80	28.20	
	At least one outdoor physical activity	71.20	28.80	
Eye specialist in last year (%)	No	75.50	24.50	<0.001
	Yes	40.60	59.40	<0.001
Handling daily work/school responsibilities (%)	Good to excellent	72.50	27.50	<0.001
	Poor to fair	65.00	35.00	<0.001

Significant association = *p* < 0.05; if 0.000 ≥ *p* < 0.001.

**Table 4:** Biological sex-based bivariate analyses to assess associations between independent variables and self-reported health care needs in relation to vision correction.

Variable	Reference	Male			Female		
		Sees Well Enough to Read without Use of Corrective Lenses	Does Not See Well Enough to Read without Use of Corrective Lenses	*p*-Value	Sees Well Enough to Read without Use of Corrective Lenses	Does Not See Well Enough to Read without Use of Corrective Lenses	*p*-Value
Biological sex (%)		75.20	24.80	<0.001	61.30	31.70	<0.001
Age (years)		Ave: 16	Ave: 16		Ave: 16	Ave: 16	0.1 or less
# ppl in residence		Ave: 8.71	Ave: 8.12		Ave: 9.13	Ave: 9.13	
Nationality (%)	UAE	77.40	22.60	<0.05	70.40	29.60	<0.001
	Other GCC countries	74.80	25.20	<0.05	79.50	20.50	<0.001
	Lebanon, Syria, Jordan, Palestine, Iraq, North Africa, Yemen, and Somalia	74.00	26.00	<0.05	69.50	30.50	<0.001
	South East Asia (India, Pakistan, Bangladesh, Sri Lanka, Philippines, and Indonesia)	69.40	30.60	<0.05	57.80	42.20	<0.001
	Europe, Canada, USA, and Australia	76.70	23.30	<0.05	62.00	38.0	<0.001
	Others and No nationality	81.50	18.50	<0.05	80.60	19.40	<0.001
Location of Residence (Emirate) (%)	Abu Dhabi	77.30	22.70	<0.001	68.90	31.10	<0.001
	Ajman	88.30	11.70	<0.001	96.70	3.30	<0.001
	Dubai	99.40	0.60	<0.001	25.70	74.30	<0.001
	Fujairah	5.10	94.90	<0.001	27.90	72.1	<0.001
	Ras Al Khaimah (RAK)	81.60	18.40	<0.001	99.80	0.20	<0.001
	Sharjah	70.60	29.40	<0.001	89.10	10.90	<0.001
	Umm Al Quwain (UAQ)	20.00	80.00	<0.001	34.90	65.10	<0.001
Parental marital status (%)	Married	75.50	24.50	<0.001	68.50	31.50	<0.001
	Separated/Divorced/Widowed, Other	73.40	26.60		67.10	32.90	
Paternal educational level (%)	Did not complete high school	79.20	20.80	<0.05	73.00	27.00	<0.001
	Completed high school	74.30	25.70	<0.05	65.80	34.20	<0.001
Maternal educational level (%)	Did not complete high school	79.10	20.90	<0.05	71.70	28.30	<0.001
	Completed high school	73.80	26.20	<0.05	64.80	35.20	<0.001
Paternal employment status (%)	Government employee	77.70	22.30		70.00	30.00	<0.001
	Private employee	72.40	27.60		63.10	36.90	<0.001
	Self employed	74.20	25.80		67.60	32.40	<0.001
	Not employed/retired	75.50	24.50		73.10	26.90	<0.001
Maternal employment status (%)	Government employee	70.10	29.90		69.40	30.60	<0.05
	Private employee	75.20	24.80		59.60	40.40	<0.05
	Self employed	77.60	22.40		68.40	31.60	<0.05
	Not employed	76.60	23.40		69.40	30.60	<0.05
Residential Ownership (%)	Own	78.40	21.60	<0.05	71.70	28.30	<0.001
	Rent	73.70	26.30	<0.05	65.30	34.70	<0.001
Household Monthly Income (%)	Less than 15,000 AED	76.10 *	23.90 *	0.1 or less	70.50	29.50	<0.05
	15,000 AED and above	72.10 *	27.90 *	0.1 or less	65.10	34.90	<0.05
Television—hrs/day (%)	Less than 1/NA	75.70	24.30		68.70	31.30	
	1 to 4	75.10	24.90		69.50	30.50	
	5 or more	75.80	24.20		66.10	33.90	
Computer/Video games—hrs/day (%)	Less than 1/NA	76.60	23.40		69.10	30.90	
	1 to 4	75.00	25.00		69.30	30.70	
	5 or more	74.90	25.10		65.90	34.10	
Mobile phone—hrs/day (%)	Less than 1/NA	73.30	26.70		70.00 *	30.00 *	0.1 or less
	1 to 4	76.10	23.90		65.50 *	34.50 *	0.1 or less
	5 or more	77.20	22.80		63.60 *	36.40 *	0.1 or less
Screen time—hrs/day (%)	Minimal	75.70	24.30		69.70	30.30	
	Mild	76.30	23.70		69.60	30.40	
	Moderate	72.80	27.20		66.90	33.10	
	High	78.00	22.00		64.90	35.10	
Outdoor physical activity (%)	No outdoor physical activity	75.00	25.00		70.20	29.80	<0.001
	At least one outdoor physical activity	75.20	24.80		67.40	32.60	<0.001
Eye specialist in last year (%)	No	77.80	22.20	<0.001	73.50	26.5	<0.001
	Yes	45.40	54.60	<0.001	38.60	61.40	<0.001
Handling daily work/school responsibility (%)	Good to excellent	76.20	23.80	<0.05	69.50	30.50	<0.05
	Poor to fair	68.20	31.80	<0.05	62.20	37.80	<0.05

* *p*-value: <0.05. Significant association = *p* < 0.05; if 0.000 ≥ *p* < 0.001.

**Table 5:** Multivariate analysis—Binary Logistic Regression Results for Self-Reported Vision Correction Needs.

Category	Variable (Reference)	Classification	Do Not See Well Enough to Read without the Use of Corrective Lenses	
			OR (Exp (B))	95% CI
Demographic	Biological sex (Male)	Female	1.042	(0.836, 1.300)
	Age		0.923	(0.834, 1.022)
	Emirate (Abu Dhabi)	Ajman	0.181 **	(0.087, 0.379)
		Dubai	2.746 **	(2.045, 3.689)
		Fujairah	9.533 **	(6.307, 14.410)
		Ras Al Khaimah (RAK)	0.129 **	(0.070, 0.236)
		Sharjah	0.711 *	(0.536, 0.944)
		Umm Al Quwain (UAQ)	7.570 **	(3.779, 15.163)
	Nationality (UAE)	Other GCC countries	1.681 *	(1.020, 2.771)
		Other Middle Eastern countries (Lebanon, Syria, Jordan, Palestine, Iraq, North Africa, Yemen, and Somalia)	1.045	(0.701, 1.559)
		South East Asia (India, Pakistan, Bangladesh, Sri Lanka, Philippines, Indonesia)	1.996 *	(1.303, 3.058)
		Europe, Canada, USA and Australia	1.864	(0.747, 4.648)
		Others and no nationality	0.584	(0.235, 1.456)
Socioeconomic	Paternal education (did not complete high school)	Completed high school	1.007	(0.775, 1.343)
	Maternal education (did not complete high school)	Completed high school	1.259	(0.946, 1.675)
	Paternal employment status (Not employed/retired)	Government employee	0.841	(0.609, 1.159)
		Private employee	0.928	(0.625, 1.378)
		Self-employed	0.853	(0.558, 1.302)
	Maternal employment status (not employed)	Government employee	1.317	(0.936, 1.854)
		Private employee	1.107	(0.752, 1.630)
		Self-employed	0.680	(0.338, 1.367)
	Home ownership (own)	Rent	0.991	(0.714, 1.376)
	Household income (less than 15,000 AED)	15,000 AED and above	1.186	(0.925, 1.522)
Behaviour and Lifestyle	Screen time (h/day) (Minimal))	Mild	1.146	(0.878, 1.495)
		Moderate	1.401 *	(1.068, 1.838)
		High	1.230	(0.876, 1.727)
Health and Function	Visiting an eye specialist in the past 12 months (No)	Yes	4.038 **	(3.051, 5.346)
	Ability to handle day to day work/school responsibilities (Good to excellent)	Poor to Fair	1.254	(0.919, 1.710)
Specificity (%)/Sensitivity (%)			93.0/45.0	
Model				
Chi-square			657.17	
*p*-value			<0.001	
Hosmer and Lemeshow test				
Chi-square			8.503	
*p*-value			0.386	

* *p*-value: <0.05. ** *p*-value: <0.001.

**Table 6:** Biological sex-based multivariate analyses—Binary Logistic Regression Results for Self-Reported Vision Correction Needs.

Category	Variable (Reference)	Classification	Male		Female	
			Do Not See Well Enough to Read without the Use of Corrective Lenses			
			OR (Exp (B))	95% CI	OR (Exp (B))	95% CI
Demographic	Age		0.796 *	(0.683, 0.928)	0.994	(0.868, 1.151)
	Emirate (Abu Dhabi)	Ajman	0.483 *	(0.235, 0.995)	0.000	(0.000, 0.000)
		Dubai	0.045 *	(0.006, 0.332)	6.346 **	(4.305, 9.356)
		Fujairah	82.833 **	(18.849, 364.023)	6.683 **	(4.161, 10.734)
		Ras Al Khaimah (RAK)	0.844	(0.420, 1.696)	0.010 **	(0.001, 0.071)
		Sharjah	1.471 *	(1.013, 2.138)	0.274 **	(0.168, 0.449)
		Umm Al Quwain (UAQ)	17.222 **	(3.348, 88.600)	6.165 **	(2.817, 13.493)
	Nationality (UAE)	Other GCC countries	2.154 *	(1.111, 4.175)	1.165	(0.439, 3.093)
		Other Middle Eastern countries (Lebanon, Syria, Jordan, Palestine, Iraq, North Africa, Yemen, and Somalia)	1.139	(0.611, 2.123)	0.908	(0.512, 1.609)
		South East Asia (India, Pakistan, Bangladesh, Sri Lanka, Philippines, Indonesia)	1.889	(0.952, 3.746)	1.860 *	(1.027, 3.368)
		Europe, Canada, USA and Australia	2.305	(0.463, 11.473)	1.861	(0.535, 6.467)
		Others and no nationality	1.386	(0.392, 4.903)	0.249 *	(0.070, 0.891)
Socioeconomic	Paternal education (did not complete high school)	Completed high school	1.073	(0.683, 1.684)	0.833	(0.556, 1.246)
	Maternal education (did not complete high school)	Completed high school	1.348	(0.850, 2.139)	1.503 *	(1.015, 2.224)
	Paternal employment status (Not employed/retired)	Government employee	0.917	(0.521, 1.613)	0.877	(0.567, 1.357)
		Private employee	1.215	(0.634, 2.332)	0.766	(0.439, 1.336)
		Self-employed	1.027	(0.495, 2.131)	0.761	(0.426, 1.360)
	Maternal employment status (not employed)	Government employee	1.388	(0.832, 2.316)	1.414	(0.854, 2.342)
		Private employee	0.930	(0.490, 1.767)	1.016	(0.599, 1.725)
		Self-employed	0.345	(0.084, 1.410)	1.025	(0.422, 2.493)
	Home ownership (own)	Rent	1.044	(0.634, 1.719)	0.981	(0.610, 1.578)
	Household income (less than 15,000 AED)	15,000 AED and above	1.428	(0.952, 2.141)	1.051	(0.747, 1.479)
Behaviour and Lifestyle	Mobile use (h/day) (less than 1/NA)	1 to 4	0.647	(0.410, 1.021)	0.983	(0.619, 1.563)
		5 or more	0.947	(0.651, 1.377)	1.060	(0.752, 1.493)
Health and Function	Visiting an eye specialist in the past 12 months (No)	Yes	4.091 **	(2.529, 6.620)	3.742 **	(2.577, 5.434)
	Ability to handle day to day work/school responsibilities (Good to excellent)	Poor to Fair	0.441 *	(0.264, 0.737)	0.987	(0.652, 1.492)
			Male	Female		
Specificity (%)/Sensitivity (%)			97.2/32.1	86.7/67.9		
Model						
Chi-square			241.661	651.190		
*p*-value			<0.001	<0.001		
Hosmer and Lemeshow test						
Chi-square			7.625	3.242		
*p*-value			0.471	0.918		

Significant association = *p* < 0.05; if 0.000 ≥ *p* < 0.001. * *p*-value: <0.05. ** *p*-value: < 0.001.

## Data Availability

The data presented in this study are available on request from the corresponding author.
